# Dental Epithelial Stem Cells Express the Developmental Regulator *Meis1*

**DOI:** 10.3389/fphys.2019.00249

**Published:** 2019-03-12

**Authors:** Maria Sanz-Navarro, Irene Delgado, Miguel Torres, Tuija Mustonen, Frederic Michon, David P. Rice

**Affiliations:** ^1^Helsinki Institute of Life Science, Institute of Biotechnology, University of Helsinki, Helsinki, Finland; ^2^Orthodontics, Department of Oral and Maxillofacial Diseases, University of Helsinki, Helsinki, Finland; ^3^Departamento de Desarrollo y Reparación Cardiovascular, Centro Nacional de Investigaciones Cardiovasculares (CNIC), Madrid, Spain; ^4^The Institute for Neurosciences of Montpellier, Inserm UMR1051, University of Montpellier, Saint Eloi Hospital, Montpellier, France; ^5^Orthodontics, Oral and Maxillofacial Diseases, Helsinki University Hospital, Helsinki, Finland

**Keywords:** mouse incisor, tooth development, palate, tongue, ectodermal organ, stem cells, SOX2, MEIS1

## Abstract

MEIS1 is a key developmental regulator of several organs and participates in stem cell maintenance in different niches. However, despite the murine continuously growing incisor being a well described model for the study of adult stem cells, *Meis1* has not been investigated in a dental context. Here, we uncover that *Meis1* expression in the tooth is confined to the epithelial compartment. Its expression arises during morphogenesis and becomes restricted to the mouse incisor epithelial stem cell niche, the labial cervical loop. *Meis1* is specifically expressed by *Sox2^+^* stem cells, which give rise to all dental epithelial cell lineages. Also, we have found that *Meis1* in the incisor is coexpressed with potential binding partner *Pbx1* during both embryonic and adult stages. Interestingly, *Meis2* is present in different areas of the forming tooth and it is not expressed by dental epithelial stem cells, suggesting different roles for these two largely homologous genes. Additionally, we have established the expression patterns of *Meis1* and *Meis2* during tongue, hair, salivary gland and palate formation. Finally, analysis of *Meis1*-null allele mice indicated that, similarly, to SOX2, MEIS1 is not essential for tooth initiation, but might have a role during adult incisor renewal.

## Introduction

Similar to human teeth, enamel cannot be regenerated in murine molars, as enamel-producing ameloblasts are lost after tooth eruption into the oral cavity. In contrast, in the mouse incisor, epithelial stem cells located in the labial cervical loop continuously produce ameloblasts and enable constant enamel renewal. This unceasing tooth growth is compensated by abrasion from gnawing. Murine dental epithelial stem cells express the transcription factor SOX2 (Juuri et al., 2012). SOX2 is required to maintain the pluripotent state of cells during early fetal development and it regulates the development of various tissues (Avilion et al., 2003; Pevny and Nicolis, 2010). *Sox2* is expressed throughout dental morphogenesis, and it marks progenitor cells that contribute to the successional molar tooth and epithelial stem cells in the incisor (Juuri et al., 2012, 2013). Loss of *Sox2* expression during dental morphogenesis leads to aberrant tooth shape, incorrect cell differentiation and, in the most extreme cases, tooth regression. In the adult incisor, lack of *Sox2* delays epithelial stem cell renewal and may trigger cellular plasticity events in order to restore a *Sox2*-expressing cell population (Sun et al., 2016; Sanz-Navarro et al., 2018).

*Meis1* gene expression is enriched in the transcriptome of *Sox2*^+^ dental epithelial cells, compared to that of murine embryonic stem cells (Sanz-Navarro et al., 2018). *Meis1* is a homeobox-containing transcription factor belonging to the Three Amino Acid Loop Extension-homeodomain (TALE) superclass and, together with *Meis2* and *Meis3*, form the *Meis* subfamily (Longobardi et al., 2014). *Meis1* is a key regulator of the development of several organs and participates in the maintenance of stem cells (Mercader et al., 1999; Hisa et al., 2004; Unnisa et al., 2012; Okumura et al., 2014; Marcos et al., 2015). Furthermore, altered expression of this gene has been linked to cancer, including leukemia and neuroblastoma (Moskow et al., 1995; Geerts et al., 2003; Argiropoulos et al., 2007). MEIS1 often acts in cooperation with HOX proteins and PBX transcription factors, however, it can also act independently. For example, in the murine embryonic trunk, MEIS1 cooperates with HOX/PBX proteins, while the role of MEIS1 in eye formation is HOX/PBX-independent (Penkov et al., 2013; Longobardi et al., 2014; Marcos et al., 2015).

To date, despite the importance of homeobox genes in tooth development (Ramanathan et al., 2018), *Meis* genes have not been described in the dental context. Here, we analyse the expression pattern of *Meis1* in the forming tooth and in the renewing mouse incisor. Additionally, we compare *Meis1* expression relative to *Meis2* and *Sox2*, and demonstrate that *Meis1* and *Sox2* are coexpressed in the incisor labial cervical loop. Finally, we show evidence that similarly, to SOX2, MEIS1 is not necessary for tooth initiation.

## Materials and Methods

### Mouse Lines

Wild type mice used for this study were from the outbred NMRI strain. *Meis1^ECFP/ECFP^* were used as *Meis1* knockout (*Meis^KO^*) (González-Lázaro et al., 2014). A total of five *Meis^KO^* embryos from four different litters were analyzed in this study, with their corresponding control littermates. The number of teeth analyzed in each experiment is specified in the text.

The stage of the embryos was determined according to the day of the vaginal plug, which was set as embryonic day 0.5 (E0.5). Postnatal day was determined from the day of birth (P0). All aspects of mouse care and experimental protocols were approved by the University of Helsinki, and the Southern Finland Council on Animal Welfare and Ethics and in accordance to the Spanish bioethical regulations for laboratory animals.

### Tissue Processing

Murine tissues were fixed in 4% paraformaldehyde (PFA) overnight at 4°C, E18.5 and adult samples were decalcified by incubating them for 2 weeks in 0.5M EDTA pH 7.5. Tissues were dehydrated in an ethanol series and embedded in paraffin. Paraffin blocks were processed into 5 μm-thick sections using a microtome.

### Histology

For histological analysis, samples were rehydrated using an ethanol series of decreasing concentration, incubated 2 min in haematoxylin, 30 s in eosin, and further dehydrated in an ethanol series, following xylene treatment and mounting. Samples were imaged with light microscopy (BX61, Olympus).

### RNAscope *in situ* Hybridization

Duplex RNAscope *in situ* hybridization (Advanced Cell Diagnostics) is a robust and sensitive method to detect single RNA molecules (Wang et al., 2012). We performed the experiments as previously described (Sanz-Navarro et al., 2018). In single-gene *in situ* RNAscope transcripts appear in red. In duplex *in situ* RNAscope, Channel 1 probes (C1) appear in blue color, while channel 2 probes (C2) are revealed in red. *Meis1*-C2 (436361-C2), *Meis2*-C1 (436371), *Sox2*-C1 (401041) and *Pbx1*-C1 (435171) probes were purchased from Advanced Cell Diagnostics. Images were obtained using a bright field microscope (BX61, Olympus). Borders of the oral and dental epithelium were drawn using an image processor.

### Immunohistochemistry

Samples were deparaffinised, rehydrated and permeabilised as previously described (Sanz-Navarro et al., 2018). Anti-MEIS1 primary antibody was used at a dilution of 1:1000 (rabbit, Abcam, ab19867), anti-SOX2 at 1:200 (goat, Santa Cruz, SC-17320) and anti-KI67 at 1:200 (rabbit, Abcam, ab16667). Secondary antibodies Alexa488 donkey anti-goat (Invitrogen) and Alexa568 goat anti-rabbit (Invitrogen) were used at 1:400. Samples were counterstained by incubating with Hoechst (1:1000, Invitrogen). Results were imaged by means of fluorescent microscopy (BX61, Olympus) and images were processed using Adobe Photoshop.

### Micro Computed Tomography (Micro-CT Scan)

Lower jaws from E14 *Meis^KO^* and control littermates were fixed in 4% PFA overnight at 4°C and incubated in a solution of 0.3% phosphotungstic acid (PTA) in 70% ethanol for 6 days. Samples were embedded into 1% low-melting point agarose and scanned with a micro-CT scanner (Bruker SKYSCAN). Reconstructions of the teeth were prepared with Avizo software (Thermo Fisher Scientific).

## Results

### *Meis1* Is Expressed in the Dental Epithelium

*Meis1* expression has been discovered in several developing organs, however, its presence has never been characterized during the development of ectodermal organs. Thus, we analyzed *Meis1* expression in the developing molar and incisor teeth with RNAscope *in situ* hybridization. Four technical replicates were used for each stage.

**FIGURE 1:**
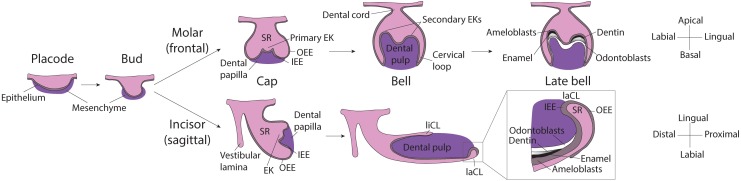
Scheme of murine molar and incisor development. **Placode stage:** At E12.5, interactions between the oral epithelium and the underlying mesenchyme cause the formation of the dental placode. **Bud stage:** At E13.5 the dental epithelium proliferates into the mesenchyme and buds. **Cap stage:** At E14.5 the primary enamel knot forms at the tip of the bud and orchestrates the proliferation of the surrounding epithelium. In the molar, the dental epithelium grows downward, while in the incisor it grows in the longitudinal axis, the expansion is led by cervical loops at the base of the tooth. The enamel organ and is composed of: stellate reticulum, stratum intermedium and enamel epithelium (inner and outer). **Molar bell and late bell stages:** In molars, at bell stage, secondary EKs mark the sites where the cusps will form. Mineralization starts from the crown and, when it reaches the cervical loops, the tooth starts to erupt and root formation starts. By this time, all enamel-producing cells are lost. **Incisor bell and late bell stages:** During bell stage, the incisor becomes asymmetrically patterned in the labial-lingual axis, the larger labial cervical loop contains stem cells that continuously generate enamel-producing ameloblasts. Enamel is deposited only on the labial side of the incisor while the lingual side is covered by dentin and cementum. *EK, Enamel knot; OEE, Outer enamel epithelium; IEE, Inner enamel epithelium; SR, Stellate reticulum; liCL, lingual cervical loop; laCL, labial cervical loop.*

Even though tooth induction events are similar for all dental types, morphogenesis of the murine molar and incisor diverges shortly after (Figure 1). For one, while the molar grows vertically, after embryonic day 14.0 (E14.0) the incisor rotates anterio-posteriorly and extends longitudinally. As the incisor elongates, the cervical loops, two bulge-like structures located at the proximal end of the tooth, become asymmetrical. The labial cervical loop enlarges and becomes the epithelial stem cell niche. Dental epithelial stem cells in the labial cervical loop give rise to rapidly dividing progenitor cells that will migrate distally toward the distal tip of the incisor and differentiate into ameloblasts (Thesleff and Tummers, 2008; Jussila and Thesleff, 2012).

**FIGURE 2:**
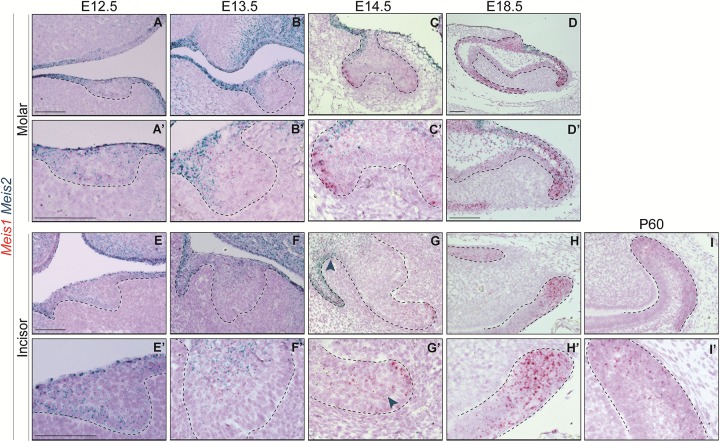
*Meis1* and *Meis2* expression patterns in molar and incisor formation. Frontal **(A–C,E)** and sagittal **(D,F–I)** sections of E12.5–E18.5 wild type mice. *Meis1* is lowly expressed in the oral and dental epithelium at placode and bud stages **(A,B,E,F)**. Its expression increases and becomes restricted to the cervical loops during cap and bell stages **(C,D,G,H)**. *Meis2* is strongly expressed in the oral epithelium and in the dental placode **(A’,E’)**. *Meis2* expression is restricted to the dental cord and vestibular lamina during the bud and cap stages **(B’,C’,F’,G’)**. During bell stage, *Meis2* is present in the proximal fraction of the molar **(D’)** but not in the incisor labial cervical loop **(H’)**. In the adult incisor (P60), *Meis1* is specifically expressed in the labial cervical loop, while *Meis2* transcripts are no longer detected in the organ **(I’)**. Scale bars 100 μm. Dashed line labels epithelial tissue.

In the course of early molar formation, a negligible amount of *Meis1* transcripts was detected in the dental placode and oral epithelium (Figure 2A,A’). This expression pattern was maintained during bud stage (Figure 2B,B’). During cap and bell stages, *Meis1* was expressed at the cervical loops (Figure 2C–D’).

**FIGURE 3:**
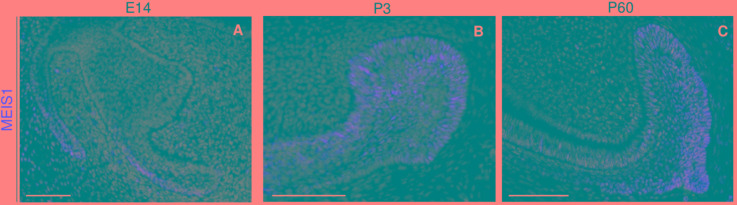
Immunodetection of MEIS1 in the forming and adult incisor. MEIS1 protein expression is detected in the incisor labial cervical loop around E14.5 **(A)**. MEIS1 expression is detected in the labial cervical loop and transit amplifying cells during postnatal day three **(B)**. During adult (P60) incisor renewal, MEIS1 specifically marks the labial cervical loop **(C)**. Scale bars 100 μm.

During the early stages of incisor morphogenesis, *Meis1* was almost undetectable in the dental epithelium (Figure 2E–F’). By cap stage, *Meis1* expression localized to the labial cervical loop (Figure 2G,G’), and its expression increased by bell stage (Figure 2H,H’). At E18.5, a few transcripts were present in the lingual cervical loop (Figure 2H). During incisor renewal (P60), *Meis1* was strongly expressed in the incisor stem cell niche (Figure 2I,I’). However, the lingual cervical loop is *Meis1-*negative (data not shown). Also, *Meis1* is expressed in the vestibular lamina throughout embryonic development. The vestibular lamina originates from the oral epithelium and generates the oral vestibule, a band located between the dentition and the lips and cheeks (Hovorakova et al., 2016). Correspondingly, MEIS1 protein was detected in the cells of the vestibular lamina and the forming labial cervical loop at E14.5 (Figure 3A). During postnatal and adult stages, majority of cells in the labial cervical loop expressed MEIS1 (Figure 3B,C). Additionally, a number of epithelial transit amplifying cells are MEIS1-positive at P3.

**FIGURE 4:**
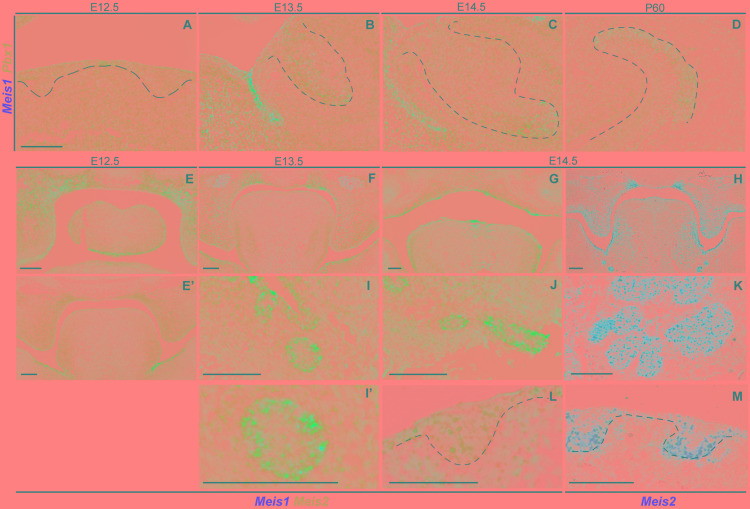
Expression of *Pbx1* in the forming incisor and *Meis1/2* expression patterns in embryonic palate, submandibular gland and hair follicle. Incisor cells expressing *Meis1* in the incisor also express *Pbx1*, as the latter is ubiquitously expressed thorough the epithelial and mesenchymal compartments during all stages of incisor development **(A–D)**. At E12.5 *Meis1* is expressed in the frontal region of the palate, both in the mesenchymal and epithelial compartments, *Meis2* is also expressed in the palatal region and in the tongue **(E)**; *Meis1* is not expressed in the posterior region of the oral cavity and *Meis2* marks the mesenchymal compartment of the palatal shelves and the tongue **(E’)**. A similar pattern is present at the posterior region of the oral cavity at E13.5 **(F)**. At E14.5 *Meis2* is expressed in both epithelial and mesenchymal compartments of the fused palate, as detected using Duplex RNAscope **(G)** and red-channel RNAscope **(H)** detection kits. *Meis1* and *Meis2* are expressed by the forming submandibular gland **(I–K)**, while the hair follicle only expresses *Meis2* at E14.5 **(L,M)**. Scale bars 100 μm. Dashed line labels epithelial tissue.

Mechanistically, MEIS1 regulates transcription by binding the DNA as a dimer, and MEIS1/PBX1 complexes are commonly found (Capellini et al., 2011; Longobardi et al., 2014). Moreover, PBX1 has been proposed to play a role in tooth patterning (Schnabel et al., 2001). We found that *Pbx1* and *Meis1* were expressed in the same cells, as *Pbx1* was ubiquitously expressed throughout the tissue at all developmental stages (Figure 4A–D).

### *Meis1* and *Meis2* Are Expressed in Different Regions of the Developing Tooth

MEIS1 and MEIS2 proteins have a similar sequence (83% identity) and an almost identical homeodomain (Oulad-Abdelghani et al., 1997). It has been proposed that they could have a redundant function during ocular lens development and neural crest cell specification (Machon et al., 2015; Antosova et al., 2016). Also, in the developing vascular system, conditional overexpression of *Meis2* can compensate for *Meis1* deficiency (Marcos et al., 2015). Therefore we were interested to see whether these two genes were coexpressed during tooth development. Four technical replicates were used for each stage.

At E12.5, *Meis2* was expressed in the oral epithelium and the molar placode, especially in the uppermost suprabasal cells (Figure 2A,A’). At the molar bud stage, *Meis2* was expressed in the oral epithelium and in the area where the dental cord will form (Figure 2B). Similarly, during cap stage, *Meis2* was expressed in the buccal side of the dental cord, the epithelium that connects the enamel organ to the oral surface (Figure 2C,C’). At E18.5, *Meis2* partially overlapped with *Meis1*, as it was expressed in the posterior region of the molar, including the cervical loop (Figure 2D,D’).

Similarly, *Meis2* was weakly expressed in the incisor placode and the surrounding oral epithelium at E12.5 (Figure 2E,E’). During bud stage, *Meis2* was expressed in the apical region of the incisor and the vestibular lamina (Figure 2F,F’). At E14.5, *Meis2* expression was mostly confined to the vestibular lamina and dental cord (Figure 2G). *Meis2* expression decreased during tooth morphogenesis, and it was undetectable in the incisor labial cervical loop during late embryogenesis and adult stages (Figure 2H,H’ and data not shown).

Altogether, *Meis2* was more strongly expressed during early tooth formation and was mostly restricted to the oral epithelium, vestibular lamina, and dental cord regions. In contrast, *Meis1* expression appeared in the vestibular lamina and cervical loops later during development. Hence, these two genes were expressed in the forming tooth with minimal overlap.

### *Meis1* and *Meis2* Are Differentially Expressed in the Forming Palate, Tongue, Hair Follicle and Submandibular Gland

Besides teeth, *Meis1* and *Meis2* were expressed in other tissues of the developing head, including the palate. Four technical replicates were used for each analyzed stage.

At E12.5 *Meis1* was expressed in the frontal region of the palate (Figure 4E). Both frontal and posterior palate expressed *Meis2* in the epithelium and, more strongly, in the mesenchyme (Figure 4E,E’). *Meis2* expression was strong throughout palatal development, as it marked the palatal shelves at E13.5 (Figure 4F) and the fused palate at E14.5 (Figure 4G,H). Also, during development, *Meis2* was expressed in the tongue mesenchyme and in the ventro-lateral region of the epithelium (Figure 4G,H).

Ectodermal organs, including teeth, have a very similar early development, and only after bud stage each organ undergoes particular morphogenesis. Thus, we investigated *Meis1* and *Meis2* expression in developing salivary glands and hair. The submandibular salivary glands expressed *Meis1* and, more strongly, *Meis2* during development (Figure 4I,K). *Meis1* expression was restricted to the epithelial fraction of the organ, while *Meis2* transcripts were identified also in the surrounding mesenchyme. The epithelial compartment of the forming hair follicle strongly expressed *Meis2*, while a few *Meis2* transcripts were expressed by the underlying mesenchyme (Figure 4L,M).

### *Meis1* and *Sox2* Are Coexpressed in the Cervical Loops During Tooth Development

As *Meis1* expression was restricted to the labial cervical loop after E14.5, we wanted to compare its expression with that of *Sox2*, a known marker for dental epithelial stem cells (Juuri et al., 2012). In a previous report, we analyzed the transcriptome of *Sox2^+^* cells at E14.5 and during adult renewal (P60), and compared them to the transcriptome of naïve murine embryonic stem cells (mESCs) (Sanz-Navarro et al., 2018). *In silico* analysis of these results (deposited at Gene Expression Omnibus, GSE104808) showed that *Meis1* expression is 29.62 fold higher in E14.5 dental *Sox2^+^* cells than in mESCs (*P*_value_ = 0.00003, ANOVA), similar to that of *Pitx2* (29.56 fold, *P*_value_ = 0.012, ANOVA), a known regulator of the incisor stem cell niche (Sun et al., 2016). Similarly, when comparing adult *Sox2^+^* dental cells and mESCs, *Meis1* was 7.34 fold enriched in the adult *Sox2^+^* cells (*P*_value_ = 0.017, ANOVA). Hence, *Meis1* was 3.93 fold enriched in embryonic *Sox2^+^* cells, compared to the adult dental epithelial cells (*P*_value_ = 0.019, ANOVA). Altogether these results indicated that at least some *Sox2^+^* cells from the dental epithelium express *Meis1*. To corroborate the results obtained with the transcriptomic analysis, we analyzed the spatial expression of these two genes by means of *in situ* hybridization. Four technical replicates were used for each analyzed stage.

**FIGURE 5:**
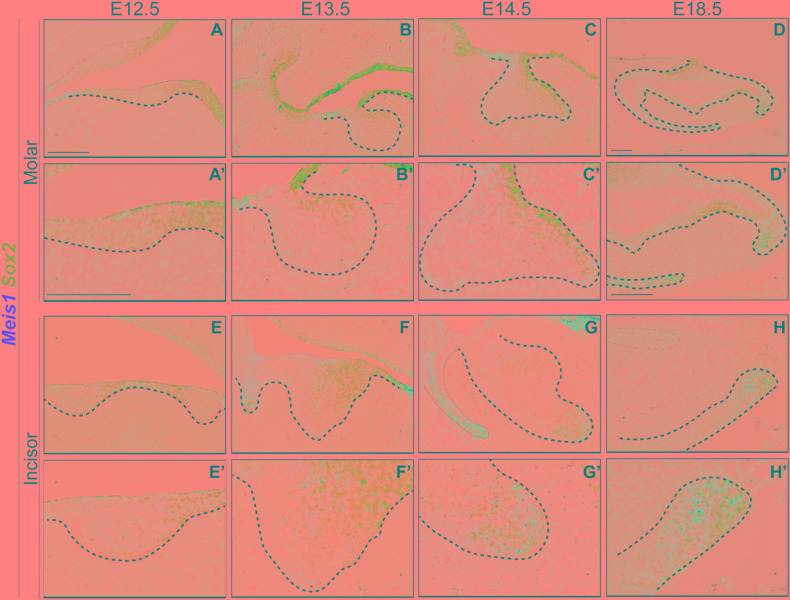
*Meis1* and *Sox2* expression patterns in developing molar and incisor. Frontal **(A–C,E)** and sagittal **(D,F–H)** sections of E12.5-E18.5 wild type mice. *Sox2* is expressed in the dental placode, mostly on the lingual side, its expression partially coincides with that of *Meis1* **(A’,E’)**. *Meis1* and *Sox2* expression partially overlaps in the dental epithelium at the bud stage. **(B’,F’)**. During cap stage, *Meis1* and *Sox2* are expressed in the developing cervical loops. Some cells from the vestibular lamina and the labial cervical loop express both MEIS1 and SOX2 **(C’,G’)**. Toward the end of tooth morphogenesis *Meis1* expression is confined to the cervical loops, in a fashion similar to that of *Sox2* **(D’–H’)**. Scale bars 100 μm. Dashed line labels epithelial tissue.

In accordance with previous reports, *Sox2* was expressed in the oral epithelium and molar placode (Juuri et al., 2012, 2013). During early morphogenesis *Sox2* expression was mostly restricted to the lingual side of the molar epithelium (Figure 5A–C’), and at E14.5 *Sox2* and *Meis1* transcripts partially overlapped (Figure 5C,C’). During late bell stage, *Sox2* expression was confined to the cervical loops, where *Meis1* transcripts were found (Figure 5D,D’). During incisor placode and bud stages, *Meis1* expression was low and scattered (Figure 5E–F’) and by E14.5 both *Meis1* and *Sox2* expression localized to the labial cervical loop (Figure 5G,G’). This pattern was maintained during late incisor morphogenesis (Figure 5H,H’). These results indicated a similar expression pattern between *Meis1* and *Sox2* during late stages of incisor and molar morphogenesis.

### *Meis1* and *Sox2* Mark the Same Cells in the Adult Incisor Labial Cervical Loop

We have discovered that after birth MEIS1 expression was maintained in the labial cervical loop, where SOX2 expression has been previously described (Juuri et al., 2012). Thus, we analyzed *Meis1* and *Sox2* expression both at the transcriptional and protein levels to determine if they mark the same cell population. Four technical replicates were used in the *in situ* RNAscope analysis and three biological replicates in the immunodetection.

**FIGURE 6:**
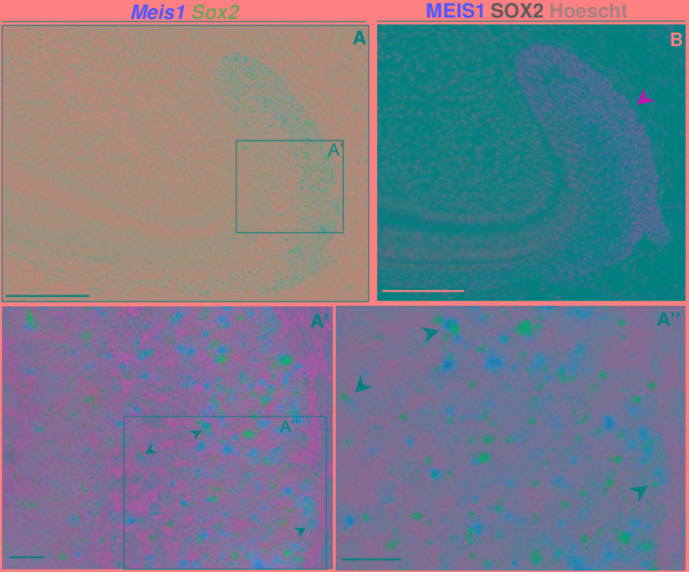
*Meis1* and *Sox2* expression in the adult incisor labial cervical loop. *Meis1* and *Sox2* are coexpressed in the incisor labial cervical loop **(A–A”)**. Black arrowheads point at cells expressing both *Meis1* and *Sox2* transcripts. In the adult labial cervical loop MEIS1 and SOX2 mark the same cells (yellow arrowhead) **(B)**. Scalebars **(A,B)**, 100 μm; **(A’,A”)**, 10 μm.

At P60, during incisor renewal, both *Meis1* and *Sox2* were expressed in the enamel epithelium and stellate reticulum of the labial cervical loop (Figure 6A). At the cell-level, we observed that a number of cells within the labial cervical loop expressed both genes (Figure 6A’,A”). MEIS1 and SOX2 protein expression completely overlapped in the adult incisor labial cervical loop (Figure 6B). Hence, adult *Sox2^+^* stem cells also expressed *Meis1*.

### *Meis1* Is Not Essential for Tooth Initiation nor for Early *Sox2* Expression

In order to comprehend the role of *Meis1* during tooth formation, and its relationship with *Sox2* expression, we utilized *Meis1*-null allele embryos (*Meis1^ECFP/ECFP^*, *Meis1^KO^*) (González-Lázaro et al., 2014).

**FIGURE 7:**
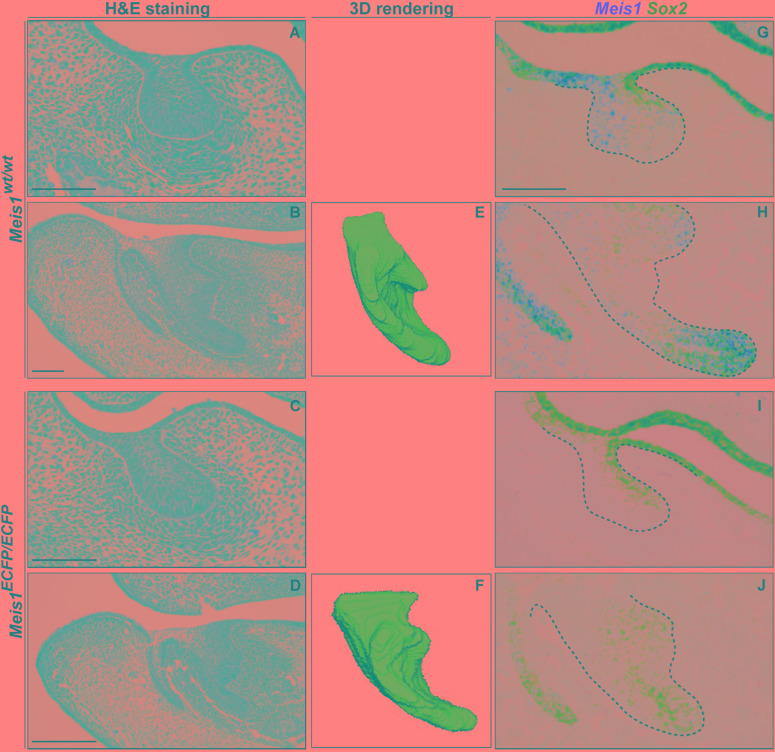
Tooth formation in *Meis1^KO^* embryos. Histological analysis show the presence of normal looking lower incisors and molars in *Meis1^KO^* embryos, similar to control littermates **(A–D)**. 3D reconstructions from micro-CT scans confirm that the forming incisors have a normal morphology **(E, F).** RNAscope *in situ* hybridization shows the complete absence of *Meis1* expression in *Meis1^KO^* embryos, *Sox2* expression pattern remains unaltered in the absence of *Meis1* expression **(G–J).** Scale bars 100 μm. Dashed line labels epithelial tissue.

Histological analysis of *Meis1^KO^* teeth indicated the presence of developing teeth at E14.5 (*N* = 4) (Figure 7A–D). 3D reconstructions of the incisors of E14.5 embryos confirmed that the morphology of the *Meis1^KO^* teeth was normal (*N* = 4) (Figure 7E,F). Also, palatal morphology was normal at E14.5 (data not shown). By means of *in situ* RNAscope we confirmed that *Meis1* expression was completely absent from E14.5 *Meis1^KO^* teeth and found that *Sox2* expression patterns was not altered in the absence of *Meis1* (*N* = 3, four technical replicates) (Figure 7G–J). Also, cell proliferation (KI67^+^ cells) and *Meis2* expression pattern remained normal (Supplementary Figure S1).

These results suggested that *Meis1* and *Meis2* did not have a redundant function in the developing tooth, and that lack of phenotype at this stage was caused by the fact that *Meis1* was only expressed in the cervical loops of the forming teeth at E14.5. Hence, *Meis1* was dispensable during the early stages of tooth development and for early *Sox2* expression. *In vivo* analysis of late developmental stages was not possible using *Meis1*-null allele mice, since they die by E14.5 (Azcoitia et al., 2005; González-Lázaro et al., 2014).

## Discussion

A complex network of genes regulate tooth morphogenesis and dental epithelial stem cell formation and maintenance (Hu et al., 2014; Krivanek et al., 2017). In this report, we have for the first time characterized the expression patterns of *Meis* genes in the forming tooth and in the renewing incisor.

During early tooth morphogenesis *Meis1* is faintly expressed throughout the dental epithelium. By E14.5, *Meis1* expression increases in the forming cervical loops of the molar and in the incisor labial cervical loop, where it is maintained during postnatal stages. *Meis2* is also expressed during early morphogenesis, but its expression disappears from the tooth as development progresses. Altogether, *Meis1* and *Meis2* expression shows minimal overlap in the tooth and absence of *Meis1* does not induce a change in the expression of *Meis2*. This suggests a different role for these two genes, in contrast to previous research (Machon et al., 2015; Marcos et al., 2015; Antosova et al., 2016). Similarly, the differential function of *Meis1* and *Meis2* in the oral cavity is supported by the fact that, while absence of *Meis2* expression leads to palatal abnormalities (Machon et al., 2015), we have not observed any palatal phenotype in the *Meis1^KO^* embryos at E14.5.

*Meis1* and *Meis2* are also differentially expressed in other ectodermal organs, such as hair, where only *Meis2* is expressed at E14.5. However, *Meis1* has been linked to the maintenance of murine epidermal hair stem cells (Okumura et al., 2014), which suggests that *Meis1* expression may appear later during hair development, in a fashion similar to the incisor.

Besides preventing epidermal stem cell differentiation in the hair bulge, *Meis1* regulates long-term haematopoietic stem cells (Unnisa et al., 2012; Okumura et al., 2014). Also, *Meis1* expression has been linked to long cell cycles and reduced cell proliferation in olfactory epithelium progenitors and cardiomyocytes (Tucker et al., 2010; Mahmoud et al., 2013). In the adult mouse incisor, we have shown that *Meis1* expression is restricted to the epithelial stem cell niche, and that it marks the *Sox2^+^* cells. Previously, we had shown that *Sox2* is important for cell differentiation and stem cell maintenance during tooth formation and adult incisor renewal (Sun et al., 2016; Sanz-Navarro et al., 2018). Thus, it is possible that *Meis1* is involved in the regulation of the dental epithelial stem cells during postnatal stages.

It is possible that MEIS1 and SOX2 are modulating each other’s expression in the dental context, as *Meis1* has been associated with the regulation of *Sox2* in different systems. In human oesophageal squamous carcinoma, *MEIS1* can suppress *SOX2* expression, while in NT2/D1 cells (neuroepithelial precursors-like cell line), *MEIS1* up-regulates *SOX3*, another member of the *SoxB1* family with overlapping functions with *Sox2* (Mojsin and Stevanovic, 2010; Rad et al., 2016; Adikusuma et al., 2017). Contrarily, in the olfactory epithelium, SOX2 downregulates MEIS1, while *Meis1* overexpression does not affect *Sox2* (Tucker et al., 2010). Moreover, MEIS1 binding sites have been described in *Sox2* enhancer regions (Marcos et al., 2015). To better understand this relationship, loss-of-function approaches would be necessary. In this study we have found a largely normal morphology in *Meis1-*null teeth at E14.5. The lack of an abnormal phenotype at this stage correlates with the late appearance of *Meis1* expression during tooth morphogenesis. However, the fact that *Meis1* is specifically expressed by *Sox2^+^* cells and the importance of MEIS1 in the regulation of other stem cell niches (Unnisa et al., 2012; Okumura et al., 2014; Miller et al., 2016; Wang et al., 2018) suggest a role for MEIS1 during later stages of incisor development or during adult renewal. The use of conditional mouse mutants during postnatal stages could lead to understand the role of MEIS1 in the incisor labial cervical loop.

To exert its function, MEIS1 binds the DNA as a dimer, like other TALE factors, and its preferred partner is PBX1 (Penkov et al., 2013; Longobardi et al., 2014). We have found ubiquitous *Pbx1* expression in the head of embryonic mice and partially overlapping with *Meis1* expression. *Pbx1* gene expression is broader than the previously reported protein pattern (Schnabel et al., 2001). This indicates that these transcripts might be exposed to extensive post-transcriptional regulation or that low levels of protein expression could not be detected. Thus, it is possible that MEIS1 and PBX1 are expressed in the same cells of the tooth and that are interacting to regulate cell behavior. However, further research is necessary in order to conclude the exact role of *Meis1* in the dental context, and its association with PBX1.

### Limitations of the Study

The early death of the *Meis1^KO^* embryos is a limitation of this study as it prevents the analysis of ameloblastic differentiation and enamel formation. Conditional loss-of-functions mouse models with longer lifespans in which *Meis1* would be specifically ablated in the dental epithelium would be a valuable tool to study the later stages of tooth morphogenesis. Alternatively, *ex vivo* culture of *Meis1^KO^* E14.5 teeth or *ex vivo* gene silencing strategies could be employed. Hence, further research is necessary to unravel the exact role of *Meis1* in the maintenance of dental epithelial stem cells.

## Conclusion

In this study, we have shown that *Meis1* and *Meis2* are expressed in different areas of the tooth, tongue, palate, hair follicle and submandibular salivary gland during morphogenesis. In the tooth, *Meis1* expression gets restricted to the cervical loops as development progresses, whereas MEIS1 and SOX2 are coexpressed in the incisor labial cervical loop during adult incisor renewal. Given the data as a whole, MEIS1 is a putative marker of dental epithelial stem cells. Altogether, *Meis1* is an interesting candidate for future studies in the fields of dental stem cells and tooth development.

## Data Availability

The datasets generated for this study are available on request to the corresponding author.

## Author Contributions

MS-N designed and performed all experiments, analyzed the data, and wrote the manuscript. ID, MT, TM, and FM provided reagents, edited the manuscript, and designed the experiments. DR designed the experiments, analyzed the data, and wrote the manuscript.

## Conflict of Interest Statement

The authors declare that the research was conducted in the absence of any commercial or financial relationships that could be construed as a potential conflict of interest.
